# Experimental modeling of hypoxia in pregnancy and early postnatal life

**DOI:** 10.2478/v10102-009-0005-3

**Published:** 2009-03

**Authors:** Mojmír Mach, Michal Dubovický, Jana Navarová, Ingrid Brucknerová, Eduard Ujházy

**Affiliations:** 1 Institute of Experimental Pharmacology & Toxicology, Slovak Academy of Sciences, Bratislava, Slovakia; 2 1^st^ Department of Pediatrics, Medical School, Comenius University, Bratislava, Slovakia

**Keywords:** hypoxia, pregnancy, animal models, behavior, antioxidants

## Abstract

The important role of equilibrium of environmental factors during the embryo-fetal period is undisputable. Women of reproductive age are increasingly exposed to various environmental risk factors such as hypoxia, prenatal viral infections, use of drugs, smoking, complications of birth or stressful life events. These early hazards represent an important risk for structural and/or functional maldevelopment of the fetus and neonates. Impairment of oxygen/energy supply during the pre- and perinatal period may affect neuronal functions and induce cell death. Thus when death of the newborn is not occurring following intrauterine hypoxia, various neurological deficits, including hyperactivity, learning disabilities, mental retardation, epilepsy, cerebral palsy, dystonia etc., may develop both in humans and in experimental animals. In our animal studies we used several approaches for modeling hypoxia in rats during pregnancy and shortly after delivery, i.e. chronic intrauterine hypoxia induced by the antiepileptic drug phenytoin, neonatal anoxia by decreased oxygen saturation in 2-day-old pups. Using these models we were able to test potential protective properties of natural (vitamin E, melatonin) and synthetic (stobadine) compounds. Based on our results, stobadine was also able to reduce hypoxia-induced hyperactivity and the antioxidant capacity of stobadine exceeded that of vitamin E and melatonin, and contrary to vitamin E, stobadine had no adverse effects on developing fetus and offspring.

## Introduction

Hypoxia during pregnancy, labor or early life stage is a major determinant of neurological morbidity and mortality in the neonatal period. Many studies have been investigating neurological deficits following perinatal hypoxia, including seizures, cerebral palsy, mental retardation, attention deficit-hyperactivity disorder, anxiety as well as other mental diseases. (Volpe, 1995; Golan *et al*., 2004; Bhat *et al*., 2005). Insufficient delivery of the tissue energy reserves (oxygen, nutrients) to the developing brain threatens its function during entire life-span up to senescence (Nyakas *et al*., 1996), and it might be one of primary factors in the pathogenesis of neurodegenerative diseases.

In the last decade the fetal origin of chronic adult diseases was proposed as the most important factor in genesis of diabetes and hypertension in adulthood. The scientists showed that malnutrition, and inadequate oxygen supply during embryofetal development may lead to the inadequate apoptosis/necrosis (do Carmo Pinho Franco *et al*., 2003, Barker, 1998) and caused maldevelopment of the organs responsible for regulation blood pressure (kidneys) and glucose (pancreas) (Barker, 1998; Bezek *et al*., 2008).

Although the understanding of perinatal asphyxia-related pathophysiology is gradually increasing, limited therapeutic options are available to prevent or even mitigate the devastating process that unfolds after injury (Brucknerová *et al*., 2008). A potential solution lies in the application of therapeutic hypothermia, protective use of antioxidants (Ujházy *et al*., 2006, Hoeger *et al*., 2006).

A study of hypoxia causing brain damage in the guinea pig was the first confirmation of the importance of fetal asphyxia (Windle and Becker, 1942). Since that time a number of research models have examined the effect of asphyxia in the fetal monkey, fetal lamb and laboratory rodents. Asphyxia has been induced by maternal hypoxemia, reduced utero-placental blood flow, umbilical cord occlusion, neonatal anoxia in 2-day old pups, while cerebral ischemia has been caused by carotid artery occlusion. (Dell'Anna *et al*., 1991; Lubec *et al*., 1997; Pulera *et al*., 1998; Spandou *et al*., 1999).

Numerous exposures to drugs or physical treatment (uterine vascular clamping, calcium channel blockers, phenytoin, cocaine, nitric oxide synthase inhibitors, chorionic villus sampling) have been shown to induce limb and central nervous system (CNS) defects in developing rats when the exposure occurs during fetal stages. Although it is a chemically and physically diverse group, exposure to each of the chemicals or events studied has been found to have vasoactive or cardioactive consequences that result in transient uteroplacental hypoperfusion (Fantel and Person, 2002).

## Hypoxia in pregnancy

Hypoxia can produce temporary brain dysfunction or permanent brain injury, depending on the duration, intensity of oxygen deprivation and age of the fetus. The hypoxia/ischemia cascade leads to neuronal cell death through overstimulation of the excitatory amino acid receptors (Monaghan *et al*., 1989; Olney, 1989), cellular calcium influx, and formation of free radicals and nitric oxide.

The results of several studies implicate that the neurotoxicity resulting from overstimulation of the excitatory amino acid receptor is extremely active in the immature rat brain compared to the adult rat brain (McDonald *et al*., 1988). Prenatal hypoxia frequently occurs during maternal convulsions in preeclampsia or eclampsia conditions. Severe asphyxia can occur in infants around the time of birth for several reasons, including compression of the umbilical cord, abruption of the placenta, abnormal uterine contractions, or failure of the neonate to successfully begin breathing. Another risk for embryo-fetus/child neurodevelopment is disruption of the milieu and integrity between mother and fetus by stress, drugs and especially the conditions leading toward excessive free radical generation.

Acute perinatal asphyxia is a major cause of death and neurological injury in newborn infants. The incidence has been estimated as 1–6 per 1000 live births and has not decreased despite advances in perinatal and obstetric care. Many asphyxiated babies die during the newborn period, and 20–30% of the survivors present with long term neurological sequelae, including spasticity, epilepsy, and mental retardation. Neurodevelopmental abnormality in childhood, presenting as seizure activity and/or motor impairment similar to that which may be observed in children with cerebral palsy, may result in the severe cases (Tuor *et al*., 1996). Milder forms of asphyxiating insults can be associated with learning disabilities and attention deficit disorders, such as the minimal brain disorder syndrome (Volpe, 1987; Hill, 1991; Carter *et al*., 1993).

## Experimental approaches

Gravity of hypoxia during pregnancy and in the early postnatal period brings us to the problem how we can improve the strategy of prevention and treatment of hypoxic-ischemic complications. Researchers developed several methodological approaches using animal models as a useful tool for elucidation of this problem. The most sensitive period for brain development is time around and shortly after delivery. From this point of view rats are suitable model. It well known that the relationship between birth and brain maturation varies substantially between species (Dobbing and Sands, 1979). The CNS of rats and mice, the most often used species for mechanistic studies in hypoxia/ischemia, is relatively immature at birth and therefore could mimic critical stages of third trimester in humans. Ligation of commom carotic artery (bilateral or unilateral) in 5–7 days old rat pups, with/without systemic oxygen decrease, is very often model for studying hypoxia/ischemia complications (Pulera *et al*., 1998; Spandou *et al*., 1999). Bilateral carotid occlusion in the 5-day-old rat, without accompanying hypoxia, causes preferential white matter injury (Uehara *et al*., 1999) with only scattered neuronal injury within the cortex. This model holds a great deal of promise for the study of mild to moderate handicap that is associated with ventriculomegaly but minimal other detectable neuropathology.

Another approach is using much younger rat pups (1–2 days old) and provokes anoxia in nitrogen atmosphere of tight sealed glass chamber. Adapting the Dell'Anna *et al*. (1991) model to the extremely immature rat has also revealed some important developmental differences in injury susceptibility (Sheldon *et al*., 1996). These extremely immature (postnatal day 1–2) animals require a longer and more severe degree of hypoxia to produce injury compared with postnatal day 7 rats, and there is a greater degree of damage to the ipsilateral subcortical developing white matter than in older rats (Sheldon *et al*., 1996).

**Figure 1:**
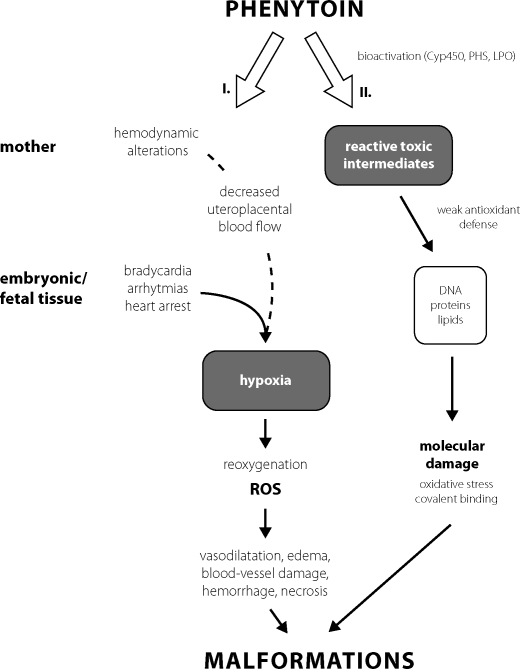
Teratogenic mechanisms of PHT. **I. Hypoxia–reoxygenation** as the cause of PHT teratogenicity, which is induced by embryonic bradycardia and arrhythmias and free radical damage during reoxygenation. Hemodynamic alterations may contribute to embryonic hypoxia (Danielsson *et al*., 1997). **II. Bioactivation of PHT by cytochrome P450 (Cyp450)**, prostaglandin H-syntase (PHS) and lipoperoxidase (LPO) to reactive toxic intermediates (Wells and Winn, 1996).

Above mentioned approaches represent acute models hypoxia/ischemia. However, hypoxia/ischemia is often caused also with chronic hypoxia-reoxygenation during maturation of organs. For this sake the model of chronic intrauterine hypoxia induced pharmacologically (phenytoin – PHT) was introduced by our lab. The proposed teratogenicity mechanism of antiseizure medication phenytoin is due to embryonic hypoxia/ischemia and production of free radicals (Danielsson *et al*., 1997; Wells and Winn, 1996; Figure 1).

## Neonatal anoxia in 2-day-old rat pups

This model represents non-invasive approach of hypoxia/ischemia study in young rat pups, what is the biggest advantage compared to carotid occlusions. It was introduced by Dell'Anna *et al*. (1991) and provides wide range of settings, i.e. temperature, age, O_2_ concentration, with minimal mother-pup unit interference. Anoxia is induced in a glass chamber with tempered bottom and cover connected to the nitrogen supply. Pups are placed into the chamber and the air inside is removed by a stream of nitrogen gas and the pups are exposed to normobaric anoxia. After anoxic insult all pups are replaced to their mothers and not surviving pups are removed.

## PHT induced chronic intrauterine hypoxia

Several mechanisms underlying PHT teratogenicity have been proposed, including disturbances in folate metabolism (Monie *et al*., 1961), vitamin K metabolism (Howe *et al*., 1995), and bioactivation of PHT to a reactive toxic intermediate (epoxide) by cytochrome P450 (Cyp450) (Martz *et al*., 1977), or co-oxidation of PHT to free radical intermediates centered in the hydantoin nucleus (Wells and Vo, 1989).

One theory suggests that PHT teratogenicity is mainly initiated by adverse pharmacological action on the embryonic heart during a sensitive stage of development, resulting in embryonic hypoxia/ischemia (Azarbayjani and Danielsson, 1998). Maternal hemodynamic alterations may contribute to embryonic hypoxia, but these alterations are not of a magnitude by which they alone could explain the observed hypoxia-related malformations (Danielson *et al*., 1992). Embryonic hypoxia has been associated with specific pathological changes such as vascular disruption, hemorrhage, and finally tissue necrosis of embryonic tissues (Danielson *et al*., 1992). The tissue necrosis, manifested as malformations in the fetus at term, may be a direct consequence of hypoxia and/or generation of ROS at reoxygenation (Figure 1).

In our experiments PHT was given to pregnant rats dissolved in deionized distilled water and pH was adjusted to 11.5 with NaOH. Buffer water with pH11.5 was used as a control vehicle. The dams could be treated by oral gavage with PHT (150 mg/kg) daily in sensitive stages of organ development or during whole pregnancy (Ujházy *et al*., 2000).

## Hypoxia induced structural and functional disturbances: possible pharmacological intervention with antioxidants

Animal models represent one and only tool for assessing disturbances during development. The observations in humans (blood draw, biochemical analysis, behavioral scans) could by make only after insult and therefore the data represents changes not mechanisms. In this matter animals are used for elucidation of mechanisms leading to origins of eclampsia, placental transfer disturbances, maternal-fetal complex communications etc. For extrapolation to humans, threshold effect and intra-/interspecies differences in the timing of developmental events must also be taken into account (Hogan and Hoel, 1989).

**Figure 2:**
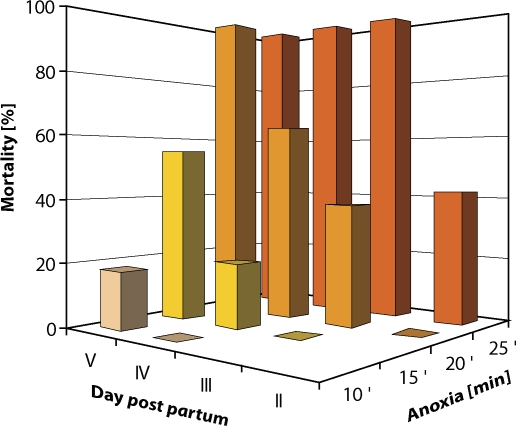
Increase of pups mortality dependent on age and duration of anoxic insult. (n=40–80 pups/group).

As we mentioned above, hypoxia represents serious risk factor in human development. Two experimental models of hypoxia (acute and chronic) were used for assessment not only structural disturbances (teratology studies) but also functional disorders (behavioral studies). Neonatal anoxia showed that 1- and 2-day old rat pups were extremely resistant to anoxic insult (Dubovický *et al*, 2000). Despite the mild disturbances after anoxia we were able to detect hyperactivity in male offspring (Ujházy *et al*., 2006). Interestingly, the synthetic pyridoindole antioxidant stobadine (STO) was able to reduce this hyperactivity, suggesting crucial role of reactive oxygen species in mechanism of anoxia induced behavioral disturbances (Ujházy *et al*., 2006). The protective effect of hypothermia following an asphyxiating insult was first demonstrated by the pioneering work of Miller (1971). Following this, several studies have confirmed the effectiveness of both intra- as well as post-asphyxic hypothermia. Similar to these findings or experiment confirmed importance of temperature in neonatal hypoxia, especially acute form. Moreower, the age factor is playing the important factor as well (Figure 2).

We studied also the effect of chronic intrauterine hypoxia produced by PHT on pre- and postnatal development of the rat offspring. When administered prenatally, PHT (inductor of hypoxia in prenatal development) was shown to exert an overwhelming effect on embryo-fetal and neuromotor development (Ujházy *et al*., 2000; Mach *et al*., 2001). It was reported that prenatal PHT treatment of rats is increasing catecholamine release in response to a mild stressor in adulthood (Makatsori *et al*., 2005). The pretreatment of pregnant rats with STO 2 h prior PHT administration partially decreased the embryotoxic effect of PHT, as manifested by increase of fetal and placental weight compared to the PHT group, and exerted a positive influence on some reproductive variables (live fetuses, resorptions, pre- and postimplantation loss) (Ujházy *et al*., 2004). The supplementation with a high dose of the natural antioxidant vitamin E (VitE) did not ameliorate the developmental toxicity of PHT and failed to protect the rat fetuses. Moreover, VitE induced growth retardation, apparent also in adulthood. Our results indicate insufficient protection of antioxidants (VitE, melatonin) in the PHT model (Mach *et al*., 2005; 2006). Prenatally administered PHT in the dose of 150 mg/kg is probably too toxic for the mother and developing fetus so that antioxidants are unable to eliminate oxidative stress. Surprisingly, we found that high doses of VitE in pregnancy, which should be safe, appear to involve a risk to the developing rat fetus due to the occurrence of slight skeletal anomalies and persistent growth retardation apparent up to adulthood (Mach *et al*., 2006).

## Conclusions

The aim of our experiments is to widen our knowledge on the possible protective effects of antioxidants in a hypoxia/ischemia models. Based on our results, the antioxidant capacity of STO exceeded that of VitE, and contrary to VitE, STO had no adverse effects on offspring. Even though STO did not fully alleviate the PHT teratogenicity, it was able to reduce PHT-induced hyperactivity and had beneficial effects on some reproductive variables. These results are indicative of a prospective use of STO as a potential protectant and a supportive therapeutic agent in pregnancies with high risk of pre-eclampsia, perinatal asphyxia or pre-term delivery, in which oxidative injury may play a crucial role.

The experimental results also demonstrating that ethological approaches in pharmacology and toxicology are an integral part of relative safety drug assessment (Dubovický *et al*., 2008). Further experiments with animal models are needed, so more accurate and precise insights into the mechanisms of hypoxia induced brain injury and sequels could be successfully treated or et least managed on the bearable level.
